# Supplementary material for: "Give me a break!" A systematic review and meta-analysis on the efficacy of micro-breaks for increasing well-being and performance
Source: PLoS One. 2022 Aug 31;17(8):e0272460. doi: 10.1371/journal.pone.0272460 (PMC9432722; doi:10.1371/journal.pone.0272460)
Supplement: S1 Table — (DOCX) [file pone.0272460.s002.docx]

**S1 Table.** **Details regarding the search string for each database.**

| Database | Search For: |
| --- | --- |
| APA PsycInfo | Abstract: work *OR* Abstract: job *OR* Abstract: organization *OR* Abstract: employee *OR* Abstract: workday) *AND* (Abstract: break *OR* Abstract:micro-break *OR* Abstract: "micro break" *OR* Abstract: pause *OR* Abstract: recovery *OR* Abstract: rest *OR* Abstract: micro-rest *OR* Abstract: "microrest" *OR* Abstract: recovery *OR* Abstract: respite *OR* Abstract: restoration *OR* Abstract: restorative *OR* Abstract: micro-restorative *OR* Abstract:"micro restorative" *OR* Abstract: "energy management") *AND* (Abstract: "well-being" *OR* Abstract: "well being" *OR* Abstract: vigor *OR* Abstract:fatigue *OR* Abstract: "job performance" *OR* Abstract: performance) *AND* Year: 1990 *To* 9999 |
| MEDLINE | ((work or job or organization or employee or workday) and (break or micro-break or "micro break" or pause or recovery or rest or micro-rest or "micro rest" or recovery or respite or restoration or restorative or micro-restorative or "micro restorative" or "energy management") and ("well-being" or "well being" or vigor or fatigue or "job performance" or performance)) Filters: Clinical Trial, Journal Article, Meta-Analysis, Randomized Controlled Trial, Review, Systematic Review, Humans, English, MEDLINE, from 1990 - 2021 |
| SCOPUS | TITLE-ABS-KEY ( work OR job OR organization OR employee OR workday ) AND TITLE-ABS-KEY ( break OR micro-break OR "micro break" OR pause OR recovery OR rest OR micro-rest OR "micro rest" OR recovery OR respite OR restoration OR restorative OR micro-restorative OR "micro restorative" OR "energy management" ) AND TITLE-ABS-KEY ( well-being OR "well being" OR vigor OR fatigue OR "job performance" OR performance ) ) AND DOCTYPE ( ar OR re ) AND PUBYEAR > 1989 AND NOT INDEX ( medline ) AND ( LIMIT-TO ( SRCTYPE , "j" ) ) AND ( LIMIT-TO ( SUBJAREA , "ENVI" ) OR LIMIT-TO ( SUBJAREA , "SOCI" ) OR LIMIT-TO ( SUBJAREA , "HEAL" ) OR LIMIT-TO ( SUBJAREA , "BUSI" ) OR LIMIT-TO ( SUBJAREA , "PSYC" ) OR LIMIT-TO ( SUBJAREA , "ARTS" ) OR LIMIT-TO ( SUBJAREA , "MULT" ) OR LIMIT-TO ( SUBJAREA , "Undefined" ) ) AND ( LIMIT-TO ( LANGUAGE , "English" ) ) AND ( EXCLUDE ( SUBJAREA , "ENGI" ) OR EXCLUDE ( SUBJAREA , "ENER" ) OR EXCLUDE ( SUBJAREA , "MEDI" ) OR EXCLUDE ( SUBJAREA , "CENG" ) OR EXCLUDE ( SUBJAREA , "CHEM" ) OR EXCLUDE ( SUBJAREA , "AGRI" ) OR EXCLUDE ( SUBJAREA , "BIOC" ) OR EXCLUDE ( SUBJAREA , "ECON" ) OR EXCLUDE ( SUBJAREA , "COMP" ) OR EXCLUDE ( SUBJAREA , "MATE" ) OR EXCLUDE ( SUBJAREA , "DECI" ) OR EXCLUDE ( SUBJAREA , "EART" ) OR EXCLUDE ( SUBJAREA , "NEUR" ) OR EXCLUDE ( SUBJAREA , "IMMU" ) OR EXCLUDE ( SUBJAREA , "NURS" ) OR EXCLUDE ( SUBJAREA , "MATH" ) OR EXCLUDE ( SUBJAREA , "PHYS" ) OR EXCLUDE ( SUBJAREA , "PHAR" ) OR EXCLUDE ( SUBJAREA , "DENT" ) OR EXCLUDE ( SUBJAREA , "VETE" ) ) |
