# Supplementary material for: "Give me a break!" A systematic review and meta-analysis on the efficacy of micro-breaks for increasing well-being and performance
Source: PLoS One. 2022 Aug 31;17(8):e0272460. doi: 10.1371/journal.pone.0272460 (PMC9432722; doi:10.1371/journal.pone.0272460)
Supplement: S2 Table — (DOCX) [file pone.0272460.s003.docx]

**S3 Table. Overall effect on fatigue and performance with the outliers included.**

| **Outcome** | **Effect size and 95% confidence interval** | | | | | | **Test of null**  **(2-Tailed)** | | **Heterogeneity** | | | | **Tau-squared** | | | |
| --- | --- | --- | --- | --- | --- | --- | --- | --- | --- | --- | --- | --- | --- | --- | --- | --- |
|  | k | d | SE | Variance | Lower limit | Upper limit | Z | p | Q | df | p | I^2^ | Tau  Squared | Standard  Error | Variance | Tau |
|  |  |  |  |  |  |  |  |  |  |  |  |  |  |  |  |  |
| Fatigue | 10 | 0.91 | 0.31 | 0.10 | 0.29 | 1.52 | 2.89 | .004 | 127.55 | 9 | .000 | 92.94 | 0.86 | 0.48 | 0.23 | 0.93 |
|  |  |  |  |  |  |  |  |  |  |  |  |  |  |  |  |  |
| Performance | 16 | 0.36 | 0.25 | 0.07 | -0.14 | 0.86 | 1.42 | .155 | 242.57 | 15 | .000 | 93.82 | 0.96 | 0.43 | 0.18 | 0.98 |
