# Supplementary material for: "Give me a break!" A systematic review and meta-analysis on the efficacy of micro-breaks for increasing well-being and performance
Source: PLoS One. 2022 Aug 31;17(8):e0272460. doi: 10.1371/journal.pone.0272460 (PMC9432722; doi:10.1371/journal.pone.0272460)
Supplement: S1 File — (DOCX) [file pone.0272460.s004.docx]

**S2 File. Detailed description of the included studies**

Studies included in the present meta-analysis were conducted in several countries across the globe, such as Japan (n = 2) [1, 2], Australia (n = 3) [3, 4, 5], Austria (n = 1) [6], Brazil (n = 1) [7], China (n = 1) [8], Netherlands (n = 1) [9], most of them taking place in the United States of America (n = 8) [10, 11, 12, 13, 14] and Germany (n = 5) [15, 16, 17, 18, 19].

Eleven studies used a sample of students [3, 4, 9, 10, 11, 13, 14, 15], whereas other ten studies used a sample of employees [1, 2, 5, 6, 7, 8, 12, 16, 18, 19] to test their experimental hypothesis. One study used “normal volunteers” to characterize its sample without clarifying which professional category these participants belong to [17]. The total number of participants in these studies was 2335, with a mean age of 31.2 years old. The employed participants are primarily white-collar employees [1, 7, 6, 12, 5, 8, 18, 19] with only one study with both blue and white-collar employees [2] working in various public and private organizations [6, 18], higher education institutions [19], Information Technology (IT) industry [8], insurance [5] or airline [7]. Only one study included in our analysis was based on a population working in Nursing Homes/Hospitals industry [16].

Most of the studies employed an experimental design, where participants were randomly allocated to different interventions (n = 15) [1, 2, 4, 6, 8, 10, 12, 13, 14, 15, 18, 19], whereas fewer used non-random allocation but with equivalent groups (n = 7) [3, 5, 7, 9, 11, 16, 17]. The majority of the studies took place in a laboratory (n = 13) [3, 4, 9, 10, 11, 13, 14, 15, 17, 19]. A smaller number was in an organizational/workplace setting (n = 9) [1, 2, 5, 6, 7, 8, 12, 16, 18]. In most studies, participants in the control group were engaged in some kind of break or free time between work tasks (n = 12) [1, 2, 5, 6, 7, 8, 9, 12, 14, 15, 16, 18], whereas in a slightly smaller number of studies these participants continued working without respite (n = 10) [3, 4, 10, 11, 13, 14, 17, 19]. If considering both the setting and the type of control, in all the studies taking place in an organization, the participants from control groups were also exposed to breaks, whereas in the laboratory studies, only in three cases they used such controls [9, 15, 14].

To study the resource replenishing effects of breaks, participants had to complete a series of tasks before taking a respite. These tasks were either relevant to organizational life, such as work simulations (n = 4) [4, 9, 10, 11] and actual work-related tasks (n = 9) [1, 2, 5, 6, 7, 8, 12, 16, 18], or irrelevant, such as various cognitive tests (n = 9) [3, 13, 14, 15, 17, 19]. Participants were exposed to different type of demands, such as cognitive (n = 7) [4, 14, 15, 17, 19], emotional (n = 3) [5, 7, 16], creative (n = 3) [3, 13], or clerical (n = 8) [1, 2, 6, 8, 9, 10, 11, 18]. To induce mental demands, several prospective memory tests were used, for example, anagrams, synonym tasks [14], or mental-addition and verification tasks [17]. We considered call center or nursing tasks as emotional demands, which are accepted in the literature as emotional labor roles, involving a higher degree of self-regulation [20, 21]. For clerical demands were considered those activities usually performed in office settings (n = 5) [1, 2, 6, 8, 18], as well as tasks closely simulating an occupation requiring visual monitoring of a computer screen, as it is usually the case in office work (n=3) [9, 10, 11]. Whereas in Bennett et al.’s [10] experiment the participants completed two rounds of the Attention Network Test (ANT) [22], two other studies used editing and typing tasks [9, 11]. Studies in which participants had to generate new ideas for different uses for a piece of paper [3] or brainstorm on how to improve their environment [13] were considered as having creative demands. Because time on task is one of the most widely studied contributors to depletion of resources during task performance [23], we considered this facet of the tasks to which participants were exposed. In our sample, for only 13 studies, the time on task was specified [3, 4, 7, 10, 11, 12, 13, 14, 16, 19], with participants spending between minimum 2 minutes [3] and maximum 4 hours [12] on resource-demanding tasks before getting a break.

Information of our independent variable — micro-breaks —, concerns the type of intervention (if it is work-related or unrelated to the task at hand), the activity performed during the break, and the break duration. In almost all of the studies, the break was a non-work one (n = 19) [1, 2, 4, 5, 6, 7, 8, 9, 11, 12, 13, 14, 15, 17, 18, 19], whereas only one study included a work-related break [16]. Two studies were included into a category with both work and non-work micro-breaks because having the same control group and pooling their results; it was impossible to differentiate between the various types of breaks participants took during these experiments [3, 10]. Thus, the low variability on this factor also impedes treating it as a moderator (despite our initial intention). Regarding what participants did during the micro-breaks, five studies used a combination of two activities, cognitive and nature-related [9, 11], cognitive and relaxation [10], cognitive and physical [17], or nature and physical [18]. For three of these studies, the combination of multiple types of activities was a result of using the same control group to compare different interventions [9, 10, 11]. For Steinborn and Huestegge’s [17] study, because there was no difference between forms of rest, as also the authors reported, we computed only one overall effect size for both activities. In Steidle et al.’s [18] study, the scholars did not analyze the two groups doing two different activities during breaks as they initially had planned. Thus only one overall effect was reported. Five studies included a cognitive micro-break, where participants were involved in activities such as watching a movie clip with negative or positive affective valence [15], watching online YouTube videos [4, 12], doing a positive reflection exercise [16], or filling in different tests or questionnaires [3]. Six studies offered a physical break activity between work bouts, where participants were engaged in various physical exercises [1, 2, 6, 7, 8, 19], whereas in other six the activities during breaks were relaxing [5, 13, 14]. Break duration varies significantly between studies, with a range between 8 seconds [14] and 10 minutes [1, 2, 5, 7, 8, 16, 18]. Hence, the heterogeneous and mixed nature of the micro-breaks employed in the included studies impedes us from exploring this aspect as a potential moderator.

The instruments used for measuring the outcomes were also considered. Well-being was usually assessed with Likert-type scales such as the Profile of Mood States [24] or Activation-Deactivation Adjective Checklist [25]. Specifically, vigor and fatigue were measured with Vigor–Activity and Fatigue–Inertia subscales of Profile of Mood States in four studies [1, 2, 5, 10], Activation-Deactivation Adjective Checklist was used in other three studies [9, 15, 12], whereas one study used a computer-based Likert scale — MoodMeter® [19]. Three-item subscale of the Utrecht Work Engagement Scale (UWES-9) [26] was also used to measure vigor in one study [18]. Fatigue was also assessed with various other instruments, such as EBF — a German standardized questionnaire [27], [6], items from Occupational Fatigue Exhaustion Recovery scale (OFER) [28], [6], items from the Chalder [29] fatigue questionnaire [7], or with one item measure in another study (i.e., “How fatigued do you currently feel?”) [18]. Thirteen studies used different objective [4, 9, 11, 14, 15, 17] and subjective [7, 8] measures of performance, as well as measures of creativity [3, 13]. We grouped accuracy (e.g., mean correct responses, number of words remembered, errors reported during completion of tasks, etc.) and reaction speed in cognitive tasks into a category of objective measures, self-perceived performance was considered as subjective measure, and creativity was considered as idea generation task performance. Specifically, accuracy was assessed as an outcome in five studies [11, 14, 15], whereas other three studies considered both accuracy and response speed [4, 9, 17]. Two studies used subjective measures for evaluating the performance of their participants [7, 8], such as a single item from the World Health Organization Health and Work Performance Questionnaire (HPQ) [30]. For measuring creativity, the mean number of responses was used as an indicator of performance in listing as many possible different uses for a piece of paper [3], or the number of nonrepetitive ideas for using a paper clip [13].

Because of the modest number of studies on each category and the low variability in many cases, we only had the methodological possibility to test a low number of moderators. For example, because of the prevalence of designs testing several different intervention types against the same control group (k = 10, 45.45% of the study sample), and low variability on certain factors, we were not able to analyze the effect of break type, or the activity performed during the breaks. For example, most studies (i.e., 19 out of 22) tested non-work-related breaks for intervention type. Similarly, we could not have tested the moderating effect of different recovery activities performed between various work tasks for our outcomes of interest. Hence, regarding the particularities of micro-breaks, the only moderator we could test remained the break duration (expressed in minutes).

Another moderator that we could analyze reflects the work/task characteristics (antecedent of the break), namely, cognitive, clerical, emotional, and creative. Since we had relatively few studies, the four types of tasks were noted evenly represented between outcomes (see Table 2 and Table 5). This categorization overlapped mainly with the task relevance (i.e., job-relevant – work simulation, or irrelevant – cognitive tests). Hence, we tested only the first one as moderator since it is the most detailed (more informative). Because the job context can influence the relationship between breaks and outcomes, we also analyzed participants’ professional category (i.e., employees vs. students) and the setting of the study (i.e., laboratory vs. workplace/organization). Moreover, we tested the role played by the control group activity (i.e., break vs. no break). Finally, we also tested if the operationalization of performance (i.e., objective performance vs. subjective performance vs. creative performance) is relevant for the effectiveness of micro-breaks.

# References

[1] Michishita R, Jiang Y, Ariyoshi D, Yoshida M, Moriyama H, Obata Y, Nagata M, Nagata T, Mori K, Yamato H. The introduction of an active rest program by workplace units improved the workplace vigor and presenteeism among workers. J Occup Environ Med. 2017 Dec 1;59(12):1140-7. doi:10.1097/jom.0000000000001121

[2] Michishita R, Jiang Y, Ariyoshi D, Yoshida M, Moriyama H, Yamato H. The practice of active rest by workplace units improves personal relationships, mental health, and physical activity among workers. J Occup Health. 2017:16-0182. doi:10.1539/joh.16-0182-OA

[3] Ellwood S, Pallier G, Snyder A, Gallate J. The incubation effect: hatching a solution?. Creat Res J. 2009 Feb 9;21(1):6-14. doi: 10.1080/10400410802633368

[4] Rees A, Wiggins MW, Helton WS, Loveday T, O'Hare D. The impact of breaks on sustained attention in a simulated, semi‐automated train control task. Appl Cogn Psychol. 2017 May;31(3):351-9. doi:10.1002/acp.3334

[5] Kennedy GA, Ball H. Power break: A brief hypnorelaxation program to reduce work-related fatigue and improve work satisfaction, productivity, and well-being. Aust J Clin Exp Hypn. 2007 Nov 1;35(2):169-93.

[6] Blasche G, Pfeffer M, Thaler H, Gollner E. Work-site health promotion of frequent computer users: comparing selected interventions. Work. 2013 Jan 1;46(3):233-41. doi: 10.3233WOR-121520

[7] Lacaze DH, Sacco ID, Rocha LE, Pereira CA, Casarotto RA. Stretching and joint mobilization exercises reduce call-center operators' musculoskeletal discomfort and fatigue. Clinics. 2010;65(7):657-62. doi:10.1590/s1807-59322010000700003

[8] Blake H, Lai B, Coman E, Houdmont J, Griffiths A. Move-It: a cluster-randomised digital worksite exercise intervention in China: outcome and process evaluation. Int J Environ Res Public Health. 2019 Jan;16(18):3451. doi:10.3390/ijerph16183451

[9] Beute F, De Kort YA. Natural resistance: Exposure to nature and self-regulation, mood, and physiology after ego-depletion. J Environ Psychol. 2014 Dec 1;40:167-78. doi: 10.1016/j.jenvp.2014.06.004

[10] Bennett AA, Gabriel AS, Calderwood C. Examining the interplay of micro-break durations and activities for employee recovery: A mixed-methods investigation. J Occup Health Psychol. 2020 Apr;25(2):126. doi: 10.1037/ocp0000168

[11] Conlin A, Hu X, Barber LK. Comparing relaxation versus mastery microbreak activity: a within-task recovery perspective. Psychol Rep. 2021 Feb;124(1):248-65. doi:10.1177/0033294119900347

[12] Janicke SH, Rieger D, Reinecke L, Connor W. Watching online videos at work: The role of positive and meaningful affect for recovery experiences and well-being at the workplace. Mass Commun Soc. 2018 May 4;21(3):345-67. doi:10.1080/15205436.2017.1381264

[13] Paulus PB, Nakui T, Putman VL, Brown VR. Effects of task instructions and brief breaks on brainstorming. Group Dyn. 2006 Sep;10(3):206. doi:10.1037/1089-2699.10.3.206

[14] Finstad K, Bink M, McDaniel M, Einstein GO. Breaks and task switches in prospective memory. Appl Cogn Psychol. 2006 Jul;20(5):705-12. doi:10.1002/acp.1223

[15] Rieger D, Reinecke L, Bente G. Media-induced recovery: The effects of positive versus negative media stimuli on recovery experience, cognitive performance, and energetic arousal. Psychol Pop Media Cult. 2017 Apr;6(2):174. doi: 10.1037/ppm0000075

[16] Clauss E, Hoppe A, O'Shea D, González Morales MG, Steidle A, Michel A. Promoting personal resources and reducing exhaustion through positive work reflection among caregivers. J Occup Health Psychol. 2018 Jan;23(1):127. doi: 10.1037/ocp0000063

[17] Steinborn MB, Huestegge L. A walk down the lane gives wings to your brain. Restorative benefits of rest breaks on cognition and self‐control. Appl Cogn Psychol. 2016 Sep;30(5):795-805. doi:10.1002/acp.3255

[18] Steidle A, Gonzalez-Morales MG, Hoppe A, Michel A, O’shea D. Energizing respites from work: a randomized controlled study on respite interventions. Eur J Work Organ Psychol. 2017 Sep 3;26(5):650-62. doi:10.1080/1359432x.2017.1348348

[19] Wollseiffen P, Ghadiri A, Scholz A, Strüder HK, Herpers R, Peters T, Schneider S. Short bouts of intensive exercise during the workday have a positive effect on neuro‐cognitive performance. Stress Health. 2016 Dec;32(5):514-23. doi:10.1002/smi.2654

[20] Delgado C, Upton D, Ranse K, Furness T, Foster K. Nurses’ resilience and the emotional labour of nursing work: An integrative review of empirical literature. Int J Nurs Stud. 2017 May 1;70:71-88. doi:10.1016/j.ijnurstu.2017.02.008

[21] Zapf D, Isic A, Bechtoldt M, Blau P. What is typical for call centre jobs? Job characteristics, and service interactions in different call centres. Eur J Work Organ Psychol. 2003 Dec 1;12(4):311-40. doi:10.1080/13594320344000183

[22] Fan J, McCandliss BD, Sommer T, Raz A, Posner MI. Testing the efficiency and independence of attentional networks. J Cogn Neurosci. 2002 Apr 1;14(3):340-7. doi: 10.1162/089892902317361886

[23] Ackerman PL, Calderwood C, Conklin EM. Task characteristics and fatigue. In: Matthews G, Desmond PA, Neubauer MC, Matthews G, Hancock PA, editors.The handbook of operator fatigue. Farnham, Surrey, UK: Ashgate Publishing Ltd.; 2012. pp. 91-101.

[24] McNair DM, Lorr M, Droppleman LF. Manual profile of mood states. San Diego, CA: Educational and Industrial Testing Services; 1971.

[25] Thayer RE. Activation-deactivation adjective check list: Current overview and structural analysis. Psychol Rep. 1986 Apr;58(2):607-14. doi: 10.2466/pr0.1986.58.2.607

[26] Schaufeli WB, Bakker AB, Salanova M. The measurement of work engagement with a short questionnaire: A cross-national study. Educ Psychol Meas. 2006 Aug;66(4):701-16. doi: 10.1177/0013164405282471

[27] Kallus KW. Erholungs-Belastungs-Fragebogen: EBF. Frankfurt: Swets Test Services; 1995.

[28] Winwood PC, Winefield AH, Dawson D, Lushington K. Development and validation of a scale to measure work-related fatigue and recovery: the Occupational Fatigue Exhaustion/Recovery Scale (OFER). Journal of Occupational and Environmental Medicine. 2005 Jun 1:594-606. doi: 10.1097/01.jom.0000161740.71049

[29] Chalder T, Berelowitz G, Pawlikowska T, Watts L, Wessely S, Wright D, et al. Development of a fatigue scale. J Psychos Res. 1993;37:147-53. doi: 10.1016/0022-3999(93)90081-P

[30] Kessler RC, Ames M, Hymel PA, Loeppke R, McKenas DK, Richling DE, et al. Using the World Health Organization Health and Work Performance Questionnaire (HPQ) to evaluate the indirect workplace costs of illness. J Occup Environ Med. 2004 Jun 1:S23-37. doi: 10.1097/01.jom.0000126683.75201.c
