# Supplementary material for: "Give me a break!" A systematic review and meta-analysis on the efficacy of micro-breaks for increasing well-being and performance
Source: PLoS One. 2022 Aug 31;17(8):e0272460. doi: 10.1371/journal.pone.0272460 (PMC9432722; doi:10.1371/journal.pone.0272460)
Supplement: Attachment — Submitted filename: Response to Reviewers.docx [file pone.0272460.s005.docx]

Ref: *PONE-D-22-07400*
Title: *Let's take a break!* *A systematic review and meta-analysis on the efficacy of micro-breaks for increasing well-being and performance*
Authors: *Patricia Albulescu, Irina Macsinga, Andrei Rusu, Coralia Sulea, Alexandra Bodnaru, Bogdan Tudor Tulbure*

RESPONSE LETTER

Dear dr. Steinborn,

Thank you for granting us the opportunity to revise and resubmit our manuscript and thank you for the feedback and extremely valuable comments for improving the quality of the paper. In the following, we will answer each and every suggestion in a point-by-point manner.

But first of all, we want to mention that when we decided on the title for the manuscript, unfortunately, we did not pay enough attention to notice that there are other published articles (even in our search results) using a similar syntagm, such we used (i.e., "Let’s have a break"), in their title (e.g., Singh, U., Ghadiri, A., Weimar, D., & Prinz, J. (2020). “Let’s take a break”: An experimental comparison of work-break interventions and their impact on performance. Journal of Business Research, 112, 128-135.).

Thus, we suggest a slight modification to the tile, by using a syntagm that seems not to be found in other article titles (at least based on a google scholar search).

Initial title: *Let's take a break! A systematic review and meta-analysis on the efficacy of micro-breaks for increasing well-being and performance*

New title: *"Give me a break!" A systematic review and meta-analysis on the efficacy of micro-breaks for increasing well-being and performance*

**Responses to the section editor’s observations**

The main point and overall impression:

As you can see, both referees seem to consider you work important overall, while at the same time provided a whole number of additional points that might be useful to consider during the revision of the manuscript. To name the most important points, both referees raised the methodical points of how to best quantify study results, and when to consider a qualitative evaluation of aspects of the studies. Here, both refer to findings on the connection between experience and performance (which sometimes go together, other times not), and, to the reliability of the underlying tests and performance measures. My personal suggestion is to also consider that there are different options to compute rest break effects and to take this into account (if necessary, in a more qualitative way by discussing it).

Overall, this work is very useful contribution of the field, and I would invite you preparing a revision of your work that addresses all points together with a cover letter that provides point-by-point replies.

<Response> Thank you for your positive feedback and the chance to revise the manuscript! As we also developed in the responses to each reviewer, the possibility to control for psychometric artefacts (reliability estimates) based on Hunter and Schmidt’s approach was limited by inconsistencies in reporting such estimates for vigor and fatigue, and with an almost total lack of reporting for the performance outcomes (with two exceptions). Hence the decision to entirely apply Borenstein et al.’s framework for the meta-analysis. But we agree that this may be an important aspect, and as you rightfully suggested, we developed and inserted in the manuscript a detailed discussion of this limitation and its implications. We also discussed and accentuated the cautionary note regarding the diversity and lack of consistency when it comes to approaches for performance and the consequential potential sources of bias. These aspects were developed (1) at the end of the Introduction (by adding new details to the already existing mentions on measurement and conceptualization issues), (2) in the Description of the included studies subsection from Results (we developed on the operationalizations being used in the included studies by critically uprising their reliability, threats to reliability and the general inconsistencies in approaches), (3) in the Discussion when presenting the results on the performance outcome as also in the Limitations subsection. This way the reader will get a stronger critical appraisal of this body of literature.

Additional requirements:

1. Please ensure that your manuscript meets PLOS ONE's style requirements, including those for file naming.

<Response> The elements related to style requirements were checked again against PlosOne’s specifications.

2. We note that you have provided funding information. However, funding information should not appear in the Acknowledgments section or other areas of your manuscript. We will only publish funding information present in the Funding Statement section of the online submission form.

Please remove any funding-related text from the manuscript and let us know how you would like to update your Funding Statement.

Please include your amended statements within your cover letter; we will change the online submission form on your behalf.

<Response> The “Financial Disclosure Statement” was removed from the manuscript. The information related to funding can be found in the “Funding Statement” document. No update is required on this funding statement.

3. We note that this manuscript is a systematic review or meta-analysis; our author guidelines therefore require that you use PRISMA guidance to help improve reporting quality of this type of study. Please upload copies of the completed PRISMA checklist as Supporting Information with a file name “PRISMA checklist”.

<Response> The filled-in PRISMA checklist was uploaded as Supporting Information the following journal guidelines (i.e., Supporting Information files should be saved as “S1_Fig.tif”, “S1_File.pdf”, etc.) as “S4_Table”. After revision, the file name was changed from “S4_Table” to “S4_Table. PRISMA Checklist”.

**Responses to the first reviewer’s comments**

Overall impression:

This is a very thorough meta-analysis on a very important topic from work and organizational research: the effects of short rest breaks on strain, motivation and perfromance. I really liked to read the paper and the quality, so far, is very impressive. However, I have some further suggestions for improvement before a publication is warranted.

<Response> Thank you for your positive feedback and appreciation!

Comments #1 to #7:

(1) When reporting I2 please use superscript for '2' => I²;

(2) p.2, l.34: "Sub-groups analyses on performance types revealed significant effects only for tasks with less cognitive demands".

(3) Overall, the data support the role of micro-breaks for well-being, while for performance, recovering from highly depleting tasks may need more than 10-minute breaks. Therefore, future studies should focus on this issue. [Or what do you mean specifcally with 'process'?)

(4) p. 5, l. 120: Please revise the sentence and refer to the study: for example

"For instance, in one study many of emplyoees reported break activities were negatively associated with increased energy (i.e., vitality) but positively related to fatigue [5]."

(5) p.13., l. 303: "The main research questions were addressed using random-effects meta-analyses based on Borenstein et al.'s [72] framework."

(6) 13, l. 306: "two-sided p-values".

(7) p.14. l329-330: τ² (Suberscript)

<Response> Thank you for the detailed observations! All these suggestions were considered and can be found modified (with track changes) in the revised manuscript.

Comment #8: p.16: 365-376: The numbers do not fully match with the data in the flow diagram: please check this.

<Response> Thank you for noticing this! We checked the information and the results of the search process. By mistake, in the text were reported 15 records instead of 25 excluded with reason (as correctly reported on the flow chart). The correction was made accordingly.

Comment #9: I missed a number of studies that might have matched with your inclusion criteria. Did you find them during search?

Singh, U., Ghadiri, A., Weimar, D., & Prinz, J. (2020). “Let’s have a break”: An experimental comparison of work-break interventions and their impact on performance. Journal of Business Research, 112, 128-135.

Blasche, G., Szabo, B., Wagner‐Menghin, M., Ekmekcioglu, C., & Gollner, E. (2018). Comparison of rest‐break interventions during a mentally demanding task. Stress and Health, 34(5), 629-638.

<Response> We found one of the suggested studies. The first study, conducted by Singh et al. (2020), was found during the systematic search but was excluded from further analysis (records excluded with reason) as it was deemed not eligible because it tested three parallel interventions with no control group to serve as a comparison (see the Comparator criterion from the PICOS – ‘Eligibility criteria’ section of the manuscript). The second study done by Blasche et al. (2018) did not appear in the initial systematic literature search. Analyzing it and applying our inclusion/exclusion criteria, we found it also not eligible as the task in which participants were engaged (from which the micro-break was taken) does not have a correspondence in the occupational setting. Moreover, as the authors conclude, “Though an academic class imposes demands also found in other kinds of mental work, and both academic classwork and office work lead to fatigue, office work and work in an academic class are obviously not identical. This warrants caution in generalizing the current findings to other contexts of mental work (p. 636)”. All other papers included in this meta-analysis used tasks with some degree of similarity with those specific to the job environment.

However, after reading again our manuscript, we noticed that the exclusion of the non-work-related studies was not made clear enough, thus in the revised version, we made this explicit mention for the eligibility criteria. Moreover, based on Blasche et al.’s (2018) suggestion we also lowered our tone on a cautionary note in the discussion of the implications of our meta-analysis for the educational setting.

Comments #10 to #12:

(10) p.21, l.385+386 should be deleted

(11) p.33, l.665 "However, at least for well-being, the answer seems to be "any type of decoupling activity" (delete comma)

(12) p.30: l.581: you write voluntary but to my impression the breaks were forced (involuntary)

<Response> All suggestions were included in the new version of the manuscript.

Comment #13: p.30, l.587 fits with that meta-analysis

Sio, U. N., & Ormerod, T. C. (2009). Does incubation enhance problem solving? A meta-analytic review. Psychological Bulletin, 135(1), 94.

<Response> We checked the suggested reference and thank you for pointing it out. Indeed, it fits with the drawn conclusion, and we included a mention based on it in the new version of the manuscript. Accordingly, the References section was also updated.

Comment #14: p.33: Another option would be adjusting for reliabilty of measure within the meta-analysis (see Schmidt-Hunter-Approach)

<Response> When we started planning the meta-analysis, we also discussed the possibility to control for psychometric artefacts based on Hunter and Schmidt’s approach. However, while screening the full-text articles and prior to beginning the coding process, we realized that there were large inconsistencies in reporting the reliabilities of the outcomes of interests. Only for some of the vigor and fatigue measures, the authors reported reliabilities (some were also one-item instruments). Moreover, for what we referred to as 'objective measures' of performance (e.g., error rates, reaction times, etc.), there was no such information. Hence, we decided to entirely apply Borenstein et al.’s framework for the meta-analysis. But we agree that this may be an important aspect, and as also per the Editor’s suggestion, we inserted in the manuscript a description and a discussion of this limitation and its implications.

**Responses to the second reviewer’s comments**

Overall impression:

The authors present an manuscript that deals with an important topic, the influence of rest breaks on feelings and performance, which is well written overall and personally interesting to me as a reader. Being a professor in personnel psychology and statistics, I will judge the manuscript with a focus on concept and methodology. See below for detailed comments.

<Response> Thank you for your positive feedback and appreciation!

Comment #1: the abstract needs revision. the abstract should include the research question, the methods and the results but in a comprehensible way. statistics or other details or specifics should not be referred to in the abstract.

<Response> The statistical details were removed from the abstract. We also reformulated the aims and methods details from the abstract in a more comprehensive way.

Comment #2: The writing is good, and the manuscript is generally interesting to read. It is also theoretically well argued, and to the point. There are some points to consider. The first concerns the instruments to measures effects of breaks, it can be a rating scale or a performance output but the effect size is difficult to compare directly, even between performance outputs of different tasks, not to speak of between feelings and performance. It depends on how good the psychometric quality of the measure is. For example, 30 min of a vigilance task deliver only a few measurement units while 10 min of a self-paced task provide a huge amount of trials to obtain reliable measures, and statistics crucially depend on it. (suggested reference: Schumann et al. (2022). Restoration of attention by rest in a multitasking world: Theory, methodology, and empirical evidence. Frontiers in Psychology, 13, 867978. doi:10.3389/fpsyg.2022.867978

<Response> Thank you for stressing this aspect! Indeed, it represents an important limitation of the current literature. As also the Editor suggested, we discussed it in several points of our manuscript. Moreover, thanks to the suggested review (Schumann et al., 2022), we also referred to some of their valuable critical insights. Precisely, (1) we began by further developing the measurement and conceptualization issues already mentioned towards the end of the Introduction, (2) continued by describing the state of the reliabilities of the used measures in the description of the sample of studies from the Results section, and (3) finally, we also addressed this issue in the Discussion by stressing out, among others, that "there are multiple methodological factors (e.g., employment of arbitrary tasks and/or unreliable or unaudited performance measures; insufficient number of trials) which could result in artefactual variance", and "even if taking into account their purpose, all these measures can be grouped under the umbrella term of performance, the many conceptual and operational differences between them could also make one rightfully argue against their aggregation. Hence, these particular results should be taken with increased caution.", as we also suggest " future studies might test the conditions under which respites positively affect subsequent performance by applying more standardized experimental paradigms and increasing consensus on methodological approaches." (These are just some examples of new mentions that we added to the manuscript.)

Comment #3: definitions of micro breaks fit with the meaning used in work psychology. I have no problem with this definition because the authors explain everything well in the manuscript, so there is no ambivalence. On the other hand, researcher with a more cognitive experimental focus often differ in the use of what is a micro break versus a short break versus long breaks. This might be discussed.

<Response> We searched in the cognitive-focused literature, and we didn’t find a clear differentiation between micro-breaks, short-breaks, and long breaks. We have cited several studies in cognitive psychology that emphasize the importance of refocusing attention after the break and we have highlighted a link between the three attention networks and breaks. We have inserted the new paragraph in the text.

Comment #4: glucose and depletion of resources. in the manuscript, the authors often refer to how task deplete mental resource in a way that gives the impression of a strong underlying basis of biology and physiology. On one occasion, for example, glucose is referred to as a causal factor determining resources. This is a difficult question, I would suggest reconsidering this point, it is in my opinion not clear.

<Response> In order to not create confusion related to the biological underlying mechanism, we deleted the entire phrase and the references.

Comment #5: tables. If possible, the tables should be presented in APA norm and maybe in a more economic way, The tables are huge and exceed a page. If the authors find ways to organise this more economically, good, otherwise, it is also okay, I would be fine with the present version.

<Response> All the included tables meet PlosOne’s style formatting requirements, which do not follow APA norms. Unfortunately, we did not find a more economical way to organize the information.
