# Supplementary material for: "Give me a break!" A systematic review and meta-analysis on the efficacy of micro-breaks for increasing well-being and performance
Source: PLoS One. 2022 Aug 31;17(8):e0272460. doi: 10.1371/journal.pone.0272460 (PMC9432722; doi:10.1371/journal.pone.0272460)
Supplement: Attachment — Submitted filename: Response to Reviewers.docx [file pone.0272460.s006.docx]

Ref: *PONE-D-22-07400R1*
Title: *"Give me a break!" A systematic review and meta-analysis on the efficacy of micro-breaks for increasing well-being and performance*
Authors: *Patricia Albulescu, Irina Macsinga, Andrei Rusu, Coralia Sulea, Alexandra Bodnaru, Bogdan Tudor Tulbure*

RESPONSE LETTER

Dear dr. Steinborn,

Thank you for giving us the opportunity to submit a revised version of the manuscript “*Give me a break!* A systematic review and meta-analysis on the efficacy of micro-breaks for increasing well-being and performance” for publication in the PLOS ONE journal. We appreciate the time and effort that you and the reviewers dedicated to providing feedback on our manuscript and are grateful for the insightful comments on and valuable improvements to our paper.

We have addressed all the new suggestions made by the reviewers. The changes are highlighted within the manuscript with track changes. Please see below, in blue, for a point-by-point response to the reviewers’ comments and concerns.

**Responses to the first reviewer’s comments**

Reviewer #1: I would like to thank the authors for revising the manuscript in line with my earlier comments. I have some few (minor) recommendation for further improvement before a publication is warranted.

<Response> Thank you for your feedback and suggestions for improvement of our manuscript. We approached every comment carefully and integrated the suggestions in the new manuscript. In the following, we also responded to each and every comment.

1) Abstract

Please inlcude specific information on the final sample size (number of studies, number of participants; i.e., data from 19 publications with 22 independent study samples, N = ...; data from p. 16) and in addition the average effect size estimates of microbreaks. This information will be helfpul for readers to get the most important results from your review in a quick way. I do agree with reviewer 2 that not all statistical information (95%CIs, p values, I²) is necessary in the abstract. But d-values and corresponding ks are 'must-haves' from my perspective.

<Response> Thank you for this suggestion. We included in the abstract a phrase summarizing the retrieved number of studies, as well as the effect sizes for each of the outcomes of interest.

2) p.16, l.380: do you mean 22 independent study samples from 19 publications?

<Response> Thank you for pointing this mistake out. The observation is correct, and we made a change here, at line 382 using the phrase “22 independent study samples” instead of the initial one, reading “22 studies”, as this, we hope, will be a clearer message.

3) Figure with PRIMSA scheme: Eligibility

Full text assessed = 43 articles

then you exclude 24

=> difference is 19

but you report 22 articles for the qualitative analysis ???

In addition, number of publications shlould be marked with k (n is usally used for participant sample size).

In the final step please report:

Included: k=19 publications with 22 independent study samples

(correct?)

<Response> Thank you for pointing this out! The changes in the Figure 1 were processed as follows:

1. Changed n with k in the entire PRISMA flow diagram
2. Articles included in the qualitative synthesis (k = 19)
3. Studies included in quantitative synthesis (meta-analysis) (k = 19 publications with 22 independent study samples)

4) p. 30, l. 575: Our results reveal that micro-break...

<Response> Thank you. We implemented the suggestion which can be found at line 577, page 30.

5) p.24, l.469: A d = 13.43 is extreme from my experience of this literature.

Have you checked effect coding and effect calculations from this specific study, maybe by different coders?

<Response> For all coding we used two independent raters. Also, all the calculations were automatically done by the software we used (Comprehensive Meta-Analysis v 3.0), based on means, standard deviations, and sample sizes as input data. However, this particular effect also puzzled us and made us check it meticulously (since human error may hay have slipped in extracting the descriptive results from the manuscript). After inspecting the data against the effect size formula, we realized that the standard deviations of each mean are very low, hence the very large d value (after the standardization of the mean difference). Bellow, we will illustrate the calculus:

Experimental group data: Mean = 2.14, Std. Dev. = 0.12, Sample size = 51

Control group data: Mean = 3.72, Std. Dev. = 0.11, Sample size = 17

d = |(M experimental – M control)| / Pooled SD

d = |(2.14 – 3.72)| / 0.118

d = 1.58 / 0.118

d = 13.39

Some observations: (1) the obtained result varies slightly from the one outputted by Comprehensive Meta-Analysis (we do not know the exact formulas behind the software, maybe include the correction for sample size or something similar); (2) the difference in means is computed in module since a lower score on fatigue (as is the case for the experimental condition compared to the control one) is the expected result; (3) the actual design of the study included 4 groups (2 experimental, the difference being the theme of the micro-break, nature exposure <n =26> or urban exposure <n = 25>, and 2 control, one with the same depleting task but without micro-break <n = 17>, and another one without any manipulation <n = 19>); since the later control fell outside our purpose (because we aimed at comparing groups who passed a similar depleting treatment, the only difference being the micro-break manipulation, as to draw conclusions on the effect of micro-breaks in working conditions), we pulled the data of the two experimental groups into one (n = 51; also these groups had similar results, i.e., means of 2.2 and 2.1) and compared it against the eligible control (n = 17). In their study, Beute and de Kort (2014), reported only the omnibus comparison between the four conditions (ANOVA analysis), without finding a significant overall effect (possible because of the small samples from each condition and the low and homogeneous variances), thus, they did not report further analyses on these data. Moreover, even for the significant differences, they reported only the overall effect size (partial etta squared) and no pairwise effects (hence the reason no similar effect size was reported in the manuscript).

6) According to your registered study protocol only control-group design studies could be included.

However, during your literature search you find that there are many studies using no control group /within-subject designs.

I agree that that this design might affect the valdity of results.

On the other hand, even the design and measures of the included studies are largely heterogenous.

So at least for the small effects sizes for performance outcomes it might be interesting to integrate and discuss some of the relevant excluded study results in the discussion. Do these studies found some performance effects?

For instance, consider tthi studies:

Engelmann, C., Schneider, M., Kirschbaum, C., Grote, G., Dingemann, J., Schoof, S., & Ure, B. M. (2011). Effects of intraoperative breaks on mental and somatic operator fatigue: a randomized clinical trial. Surgical endoscopy, 25(4), 1245–1250. https://doi.org/10.1007/s00464-010-1350-1

Engelmann, C., Schneider, M., Grote, G., Kirschbaum, C., Dingemann, J., Osthaus, A., & Ure, B. (2012). Work breaks during minimally invasive surgery in children: patient benefits and surgeon's perceptions. European journal of pediatric surgery : official journal of Austrian Association of Pediatric Surgery ... [et al] = Zeitschrift fur Kinderchirurgie, 22(6), 439–444. https://doi.org/10.1055/s-0032-1322542

Park, A. E., Zahiri, H. R., Hallbeck, M. S., Augenstein, V., Sutton, E., Yu, D., Lowndes, B. R., & Bingener, J. (2017). Intraoperative "Micro Breaks" With Targeted Stretching Enhance Surgeon Physical Function and Mental Focus: A Multicenter Cohort Study. Annals of surgery, 265(2), 340–346. https://doi.org/10.1097/SLA.0000000000001665

It is hard to conduct real intervention-control-group designs in such work settings. For instance, consider the Engelmann studies that showed real performance effects in a way that breaks improved patient outcomes (not only the concentration of the surgeons).

However, such studies found positive effects of breaks on performance which bolster the argument that breaks might even improve perfomance. Another argument is also that in many rest break studies the intervention group with additional rest breaks has actually a shorter total time on task (or work duration). Thus, significant increases in work performance (if this is assessed) are not really to be expected, it will be fine if this might be similar to the control group which has a longer time on task.

In sum, I will prefer if you could add some additional insights relating to performance effects from the excluded study types into the discussion.

<Response> Thank you for this suggestion! We further developed the discussion, looking at all the studies on performance from the 43 papers included in the eligibility assessing phase (including the ones that you suggested). Hence, we added a more extensive overview on the effect of breaks on performance (see Pages 33-34, lines 662-694). Because we included papers not cited previously throughout the manuscript, the in-text citations and reference list were also updated.

**Responses to the second reviewer’s comments**

Reviewer #2: The manuscript has improved greatly, and I would recommend this work for publication. I have no further comments.

<Response> Thank you for all the suggestions made during the first round of feedback, which made it possible to have this new and improved version of the manuscript. Also, thank you for your kind support during this review process.
